# Attentional conditions differentially affect early, intermediate and late neural responses to fearful and neutral faces

**DOI:** 10.1093/scan/nsaa098

**Published:** 2020-07-23

**Authors:** Sebastian Schindler, Maximilian Bruchmann, Anna-Lena Steinweg, Robert Moeck, Thomas Straube

**Affiliations:** Institute of Medical Psychology and Systems Neuroscience, University of Muenster, Münster D-48149, Germany; Otto Creutzfeldt Center for Cognitive and Behavioral Neuroscience, University of Muenster, Münster D-48149, Germany; Institute of Medical Psychology and Systems Neuroscience, University of Muenster, Münster D-48149, Germany; Otto Creutzfeldt Center for Cognitive and Behavioral Neuroscience, University of Muenster, Münster D-48149, Germany; Institute of Medical Psychology and Systems Neuroscience, University of Muenster, Münster D-48149, Germany; Institute of Medical Psychology and Systems Neuroscience, University of Muenster, Münster D-48149, Germany; Institute of Medical Psychology and Systems Neuroscience, University of Muenster, Münster D-48149, Germany; Otto Creutzfeldt Center for Cognitive and Behavioral Neuroscience, University of Muenster, Münster D-48149, Germany

**Keywords:** emotional expressionattention taskline, gender/sex or emotion discriminationfeature-based attentionEEG/ERP

## Abstract

The processing of fearful facial expressions is prioritized by the human brain. This priority is maintained across various information processing stages as evident in early, intermediate and late components of event-related potentials (ERPs). However, emotional modulations are inconsistently reported for these different processing stages. In this pre-registered study, we investigated how feature-based attention differentially affects ERPs to fearful and neutral faces in 40 participants. The tasks required the participants to discriminate either the orientation of lines overlaid onto the face, the sex of the face or the face’s emotional expression, increasing attention to emotion-related features. We found main effects of emotion for the N170, early posterior negativity (EPN) and late positive potential (LPP). While N170 emotional modulations were task-independent, interactions of emotion and task were observed for the EPN and LPP. While EPN emotion effects were found in the sex and emotion tasks, the LPP emotion effect was mainly driven by the emotion task. This study shows that early responses to fearful faces are task-independent (N170) and likely based on low-level and configural information while during later processing stages, attention to the face (EPN) or—more specifically—to the face’s emotional expression (LPP) is crucial for reliable amplified processing of emotional faces.

## Introduction

Emotional facial expressions constitute a significant part of communication as they transfer crucial non-verbal signals to others. Therefore, their processing is assumed to be prioritized when compared to neutral facial expressions. In line with this assumption, amplifications of early, intermediate and late event-related potentials (ERPs) have been reported especially for expressions signalling threat or danger. The occipitally P1 component reflects early stages of stimulus detection and discrimination (Luck and Hillyard, 1994; Hopfinger and Mangun, 1998; Vogel and Luck, 2000) and is strongly driven by low-level influences (Rossion and Caharel, 2011; Schindler *et al.,* 2019b). Findings on how fearful faces modulate the P1 are mixed, with some studies reporting larger amplitudes for emotional compared to neutral expressions (e.g. see Mühlberger *et al.,* 2009; Foti *et al.,* 2010; Müller-Bardorff *et al.,* 2018), while others do not find such an effect (e.g. see MacNamara *et al.,* 2011; Wieser *et al.,* 2012; Smith *et al.,* 2013). The N170 is viewed as a structural and configural encoding component (Eimer, 2011) and found to be reliably modulated by fearful expressions (Hinojosa *et al.,* 2015). The subsequent early posterior negativity (EPN) has previously been related to early attentional selection processes (Schupp *et al.,* 2004; Wieser *et al.,* 2010). It has been observed as a differential negativity in studies contrasting emotional with neutral expressions (Schupp *et al.,* 2004; Schindler *et al.,* 2017). Several studies have reported its enlargement for fearful stimuli (e.g. see Walentowska and Wronka, 2012; Wieser *et al.,* 2012; Schindler *et al.,* 2019a), while in other studies no differences between fearful and neutral faces were observed (e.g. see Santos *et al.,* 2008; Herbert *et al.,* 2013). Finally, the late positive potential (LPP) is indicative of controlled attentional processes and stimulus evaluation (Schupp *et al.,* 2006; Hajcak *et al.,* 2009), particularly when the appraisal of affective meaning is involved (Schupp *et al.,* 2006; Wessing *et al.,* 2013). With respect to emotional facial stimuli, some studies have found fearful faces to elicit larger late positivities than neutral faces (e.g. see Wieser *et al.,* 2012; Herbert *et al.,* 2013; Grunewald *et al.,* 2019), but others do show no differential effects (e.g. see Peltola *et al.,* 2018; Schindler *et al.,* 2019a).

The attentional focus might be an underlying mechanism to explain the inconsistent findings regarding emotional ERP effects. Here, attentional instructions should amplify the emotional modulation of ERPs involved in the assumed processing stage. It is expected that early components should show a higher automaticity of emotional amplification while later components should predominantly depend on the attentional condition and its instruction (for a review, see Schindler and Bublatzky, 2020). A mechanistic explanation for this pattern would be that at early stages, emotion effects are mainly based either on feedforward processes within the visual cortex (Rossion and Caharel, 2011) or on rapid local feedback loops or rapid initial amygdala feedback (e.g. see Müller-Bardorff *et al.,* 2018). In contrast, the LPP is hypothesized to reflect the activation of broad occipitoparietal regions (Sabatinelli *et al.,* 2007, 2014; Liu *et al.,* 2012) linked to higher cognition, such as stimulus evaluation and affective labelling (Schupp *et al.,* 2006; Hajcak *et al.,* 2009). Here, both emotional feedback from the amygdala and top-down signalling from frontoparietal attention networks might synergistically increase the processing of emotional stimuli (Pourtois *et al.,* 2013). As these later processes are more vulnerable to competing tasks (e.g. see Schupp *et al.,* 2007), they should only be affected by emotion if cognitive resources are available.

For the P1, preliminary evidence indicates that enlarged amplitudes for fearful faces are more often reported when faces serve as distracters, e.g. when an overlaid object has to be discriminated (e.g. Santos *et al.,* 2008). Distraction tasks also seem to attenuate or abolish emotion effects for the consecutive components leading to absent N170 (Santos *et al.,* 2008; Framorando *et al.,* 2018; Li *et al.,* 2018) or EPN modulations for fearful or angry expressions (Li *et al.,* 2018; Wu *et al.,* 2019). For the LPP, emotion effects for threatening faces seem to depend even more heavily on specific task sets. Several studies observe no significant differences during perceptual tasks (Müller-Bardorff *et al.,* 2016), passive viewing tasks (Peltola *et al.,* 2018) or tasks directing attention to the face but not to the expression (Syrjänen *et al.,* 2018).

Among the many studies comparing specific tasks (e.g. Neath-Tavares and Itier, 2016; Wu *et al.,* 2019), there are—to the best of our knowledge—only four studies to have realized a design with more than two task conditions (Rellecke *et al.,* 2012; Valdés-Conroy *et al.,* 2014;Itier and Neath-Tavares, 2017 ; Acunzo *et al.,* 2019). Moreover, as some studies focused only on early ERPs (Itier and Neath-Tavares, 2017; Acunzo *et al.,* 2019), the picture which these studies draw has remained rather incomplete. Overall, P1 emotion effects remain inconclusive (reporting task-independent effects, Rellecke *et al.,* 2012; Valdés-Conroy *et al.,* 2014; reporting a lack of task-independent effects, Itier and Neath-Tavares, 2017; Acunzo *et al.,* 2019), while emotion effects are consistently found for the N170 and EPN, being either task-insensitive (Rellecke *et al.,* 2012; Itier and Neath-Tavares, 2017) or task-modulated (N170, Valdés-Conroy *et al.,* 2014). Surprisingly, analyses of the LPP provide conflicting interpretations, with one study reporting emotion effects are strongest during an emotion decision task (Rellecke *et al.,* 2012) and the other study not supporting this finding (Valdés-Conroy *et al.,* 2014). Taken together, a clear picture of task-dependent emotion effects cannot be derived from the literature, and studies are needed which systematically vary task instructions to directly test how neural responses across all relevant time windows depend on the attended feature.

## The current design and hypothesis

In this pre-registered study (https://osf.io/qgwzd), we investigated feature-based attention effects on early (P1, N170), mid-latency (EPN) and late (LPP) processing stages for fearful *vs* neutral faces. To this end, participants (*N* = 40) were presented fearful and neutral faces, always displayed with an overlay of thin horizontal or vertical lines. We used three attention tasks to gradually increase the attention to emotionally relevant features of the facial stimuli (line discrimination, sex discrimination, facial expression discrimination). Based on the line of argumentation outlined above, we predicted that the later the component of the ERP, the higher the relevance of the attentional focus on emotionally relevant features for finding emotional modulations of these components. In particular, we expected the emotional P1 modulation to be strongest in the perceptual task, while the N170 and especially the EPN should show stronger emotion effects in the sex and emotion decision task. LPP emotion effects were expected only in the emotion decision task.

## Methods

### Participants

In total, 42 participants were recruited from the University of Münster. They all gave written informed consent and received 10 euros per hour for participation. One participant was excluded due to a neurological disorder and one due to noisy EEG. According to the registered data sampling plan, this led to a final sample of 40 participants, for which power calculations using G^*^Power 3.1.7 (Faul *et al.,* 2009) showed a power of >90% to detect medium effect sizes (*f* = 0.25). The resulting 40 participants (30 female) were 23.33 years old (*s.d.* = 3.08) on average. All participants had normal or corrected-to-normal eye vision, were right-handed and had no reported history of neurological or psychiatric disorders.

### Stimuli

The facial stimuli were taken from the Radboud Faces Database, exhibiting well-standardized eye position and head orientation (Langner *et al.,* 2010). Cut-out grey-scaled faces of 32 identities (16 male and 16 female), depicting neutral and fearful expressions, were chosen from this database. The faces were shown with an overlay of five thin horizontal or vertical lines, displayed within the boundaries of the face (horizontal lines 1.7 length; vertical lines 2.3 length; thickness 0.01; centred around *x* = 0.1, *y* = −0.1).

### Procedure

While participants were prepared for the EEG, they responded to a demographic questionnaire as well as to the BDI-II and STAI Trait questionnaire (Spielberger *et al.,* 1999; Hautzinger *et al.,* 2009) and to a short version of the NEO-FFI (Körner *et al.,* 2008). Participants were seated 60 cm in front of a gamma-corrected display (NBC Multisync E231W 23″) running at 60 Hz with a Michelson contrast of 0.9979 (*L*_min_ = 0.35 cd/m^2^; *L*_max_ = 327.43 cd/m^2^). The background was set to medium grey (RGB 128, 128, 128). Participants were instructed to avoid eye movements and blinks during stimulus presentation. Participants started either with the perceptual decision, the sex decision or the emotion decision task, while task order and response buttons (x and m) were counterbalanced. In each trial, participants were confronted with a two-alternative forced choice task and had to decide whether the overlaid line orientation was horizontal or vertical, whether the sex was male or female or whether the expression was fearful or neutral. In all tasks, trial structure and stimuli were identical. Each trial started with the presentation of a fixation cross for 800–1000 ms after which a face was displayed for 100 ms. The face’s display was followed by the presentation of another fixation cross for 1500 ms during which responses were recorded. Each face was repeated twice for a total of 64 fearful and 64 neutral faces presented in each task condition, summing up to a total of 384 trials. Of note, each identity was in total repeated six times with a neutral and six times with a fearful expression.

### E‌EG recording and pre-processing

EEG signals were recorded from 64 BioSemi active electrodes using BioSemi’s Actiview software (www.biosemi.com). Four additional electrodes measured horizontal and vertical eye movements. Recording sampling rate was at 512 Hz. Offline data were re-referenced to average reference and filtered with a high-pass forward filter of 0.01 (6 db/oct) as well as a 40 Hz low-pass zero-phase filter (24 db/oct). Recorded eye movements were corrected using the automatic eye-artefact correction method implemented in BESA (Ille *et al.,* 2002). Remaining artefacts were rejected based on absolute threshold (120 μV), gradient (75) and low signal change (0.01). Noisy EEG sensors were interpolated using a spline interpolation procedure. The stimuli on the liquid crystal display (LCD) display in use were found to have a trigger delay of 15 ms, as measured by a photodiode. This delay was corrected during epoching. Filtered data were segmented from 200 ms before stimulus onset to 1000 ms after stimulus presentation. Baseline correction used the 200 ms before stimulus onset. On average, 5.29 electrodes were interpolated and 23.17% trials were rejected. On average this resulted in 50 fearful and 50 neutral faces kept for the perceptual, 49 fearful and 49 neutral trials kept for the sex and 48 fearful and 49 neutral faces kept for the emotion task across participants. For kept trials, no main effect of emotion (*F*_(1,39)_ = 0.06, *P* = 0.814, η_P_^2^ = 0.001) and of task (*F*_(2,78)_ = 0.55, *P* = 0.579, η_P_^2^ = 0.014) and no interaction were found (*F*_(2,78)_ = 0.85, *P* = 0.430, η_P_^2^ = 0.021).

### Data analyses

All data were analysed using two (emotion, fearful, neutral) by three (task, perceptual, sex, emotion) repeated measures analyses of variance (ANOVAs). For analyses of the P1, N170 and EPN, laterality (left/right) was included as a factor. Partial eta-squared (η_P_^2^) was used to describe effect sizes (Cohen, 1988). The pre-condition of sphericity was tested using Mauchly’s test of sphericity, and in case of a violation, degrees of freedom were corrected in accordance with Greenhouse–Geisser. For behavioural data, reaction times above 100 ms and below 1500 ms were regarded as correct responses (‘hit’). Please note that for two participants, responses were re-coded in one task condition (sex and perceptual task).

EEG scalp data were statistically analysed using EMEGS (Peyk *et al.,* 2011). Time windows were segmented into intervals from 80 to 100 ms for the P1, from 130 to 170 ms for the N170, from 250 to 350 ms for the EPN and from 400 to 600 ms for the LPP. Based on our registration, we measured the P1 and N170 over two symmetrical occipital clusters (left P9, P7, PO7; right P10, P8, PO8), the EPN over temporo-occipitally clusters (left P9, P7, TP7, T7; right P10, P8, TP8, T8) and the LPP over a centroparietal cluster (CP3, CP1, CPz, CP2, CP4, P3, P1, Pz, P2, P4). Analyses of covariance (ANCOVAs) with reaction time as a covariate were calculated to account for possible influences of reaction time differences on ERP modulations. We therefore corrected reaction time effects on ERP amplitudes by entering the respective RT data per condition as a within-subject covariate (as implemented in ezANOVA from the R-package ‘ez’; see Lawrence and Lawrence, 2016). Finally, we tested average absolute activation in horizontal and vertical EOG channels, using repeated measures ANOVAs. The pre-registration can be retrieved from the Open Science Framework (https://osf.io/qgwzd).

## Results

### Behavioural results

**Fig. 1:**
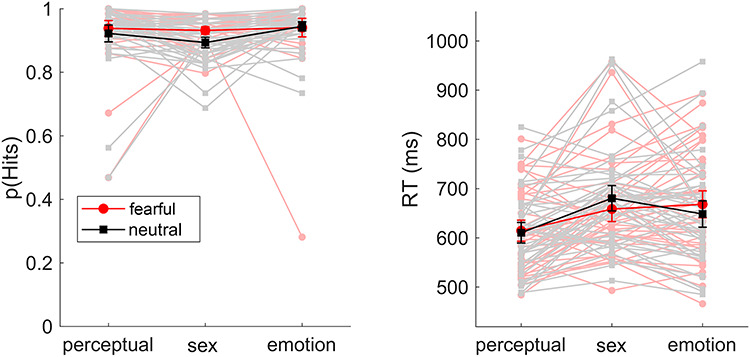
Hit rate (in percent) and reaction time (in milliseconds) results across the three attention tasks. Every subject is displayed; means across subjects are highlighted in dark printed in bold form. Error bars represent 95% confidence intervals.

Regarding hit rate, there was little evidence that the number of correct choices was affected by emotion (*F*_(1,39)_ = 3.68, *P* = 0.062, η_P_^2^ = 0.086) and no significant effect of task (*F*_(1.68,65.35)_ = 1.95, *P* = 0.158, η_P_^2^ = 0.047) and no interaction were found (*F*_(1.47,57.49)_ = 2.66, *P* = 0.094, η_P_^2^ = 0.064). Regarding reaction time, no main effect of emotion (*F*_(1,39)_ = 0.03, *P* = 0.870, η_P_^2^ = 0.001) but a main task effect was identified (*F*_(2,78)_ = 11.03, *P* < 0.001, η_P_^2^ = 0.221). Here, reaction times were significantly shorter in the perceptual task than in the sex (*P* < 0.001) and in the emotion task (*P* = 0.001), the latter two not differing (*P* = 0.395). In addition, a significant emotion × task interaction effect was found (*F*_(2,78)_ = 12.71, *P* = 0.001, partial η^2^ = 0.246). *Post hoc* test showed that compared to the perceptual task, larger emotion effects were found in the sex (*P* = 0.002) and emotion tasks (*P* < 0.001), the latter two not differing (*P* = 0.096). Here, fast responses to fearful expressions were found in the sex task and slower responses in the emotion task (see Figure 1).

### ERP results

#### P1

**Fig. 2:**
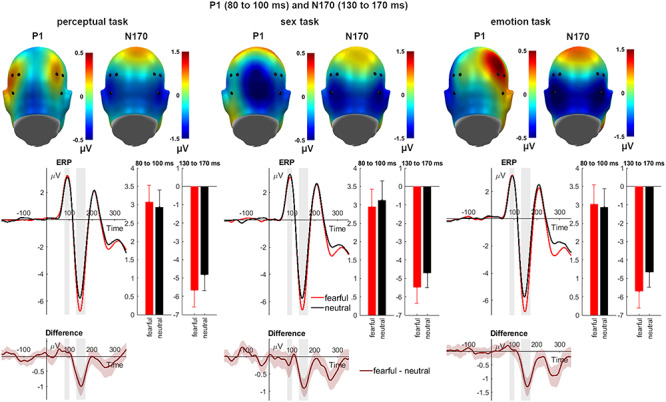
Main effects of emotional expression on P1 and N170 amplitudes. Scalp topographies depict the differences between fearful and neutral expressions. ERP waveforms show the time course over highlighted sensors. Respective difference plots contain 95% bootstrap confidence intervals of intra-individual differences. For bar charts, error bars show 95% confidence intervals.

With respect to the P1 component, no main effect of emotion (*F*_(1,39)_ = 0.02, *P* = 0.883, η_P_^2^ = 0.001; see Figure 2) and no main effect of task (*F*_(2,78)_ = 0.24, *P* = 0.787, η_P_^2^ = 0.006) or of channel group could be identified (*F*_(1,39)_ = 0.60, *P* = 0.444, η_P_^2^ = 0.015). There was no interaction of emotion and task (*F*_(2,78)_ = 2.19, *P* = 0.119, η_P_^2^ = 0.053). Analyses for each task separately showed no significant emotion effect in the perceptual (*F*_(1,39)_ = 2.15, *P* = 0.151, partial η^2^ = 0.052), the sex (*F*_(1,39)_ = 1.31, *P* = 0.260, partial η^2^ = 0.032) and the emotion task (*F*_(1,39)_ = 0.75, *P* = 0.391, partial η^2^ = 0.019). Further interactions remained insignificant as well (*Fs* < 2.13, *P*s > 0.126).

#### N170

Regarding the N170, there was a large main effect of emotion (*F*_(1,39)_ = 90.81, *P* < 0.001, η_P_^2^ = 0.700; see Figure 2) but no main effect of task (*F*_(2,78)_ = 0.91, *P* = 0.405, η_P_^2^ = 0.023). Another significant main effect could be found for the channel group (*F*_(1,39)_ = 6.75, *P* = 0.013, η_P_^2^ = 0.148). Regarding these significant main effects, fearful faces elicited larger N170 amplitudes than neutral ones, and larger N170 amplitudes were recorded over the right compared to the left electrode cluster. There was no interaction of emotion and task (*F*_(2,78)_ = 1.73, *P* = 0.184, η_P_^2^ = 0.043) and all further interactions remained insignificant as well (*Fs* < 0.36, *P*s > 0.702).

#### Early posterior negativity

**Fig. 3:**
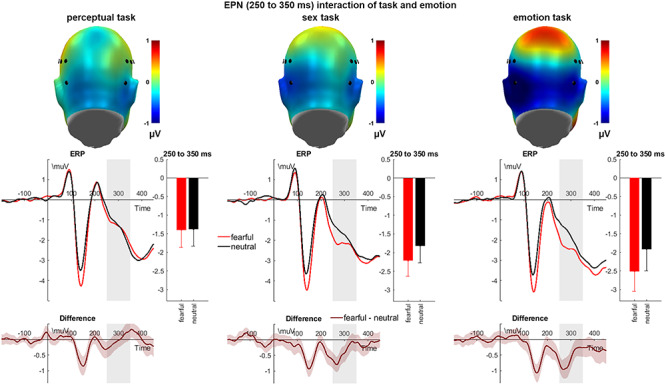
Interaction effects of emotional expression and task condition on the EPN. Scalp topographies depict the amplitude differences between fearful and neutral expressions. ERP waveforms show the time course over highlighted sensors. Respective difference plots contain 95% bootstrap confidence intervals of intra-individual differences. For bar charts, error bars show 95% confidence intervals.

For the EPN, both main effects of emotion (*F*_(1,39)_ = 14.59, *P* < 0.001, η_P_^2^ = 0.272; see Figure 3) and task reached significance (*F*_(2,78)_ = 9.37, *P* < 0.001, η_P_^2^ = 0.194), but no effect of channel group was found (*F*_(1,39)_ = 1.18, *P* = 0.283, η_P_^2^ = 0.029). Fearful faces elicited larger EPN amplitudes than neutral ones, and both the sex and the emotion tasks led to larger EPN amplitudes than the perceptual task (*P*s = 0.001). The amplitudes in the sex and the emotion tasks did not differ from one another (*P* = 0.377).

As expected, we observed a significant interaction of emotion and task on the EPN amplitude (*F*_(2,78)_ = 7.13, *P* = 0.001, η_P_^2^ = 0.155; see Figure 3). *Post hoc* tests show that compared to the perceptual task, larger emotion effects were found in the sex task (*P* = 0.016) and in the emotion task (*P* = 0.001), the latter two not differing (*P* = 0.179). There was an interaction of task and channel group (*F*_(2,78)_ = 4.12, *P* = 0.020, η_P_^2^ = 0.096), showing no task differences over the left (*F*_(1.49,57.95)_ = 1.55, *P* = 0.223, η_P_^2^ = 0.038) but significant effects over the right electrode cluster (*F*_(2,78)_ = 16.90, *P* < 0.001, η_P_^2^ = 0.302). All further interactions were insignificant (*Fs* < 1.02, *P*s > 0.354).

#### Late positive potential

**Fig. 4:**
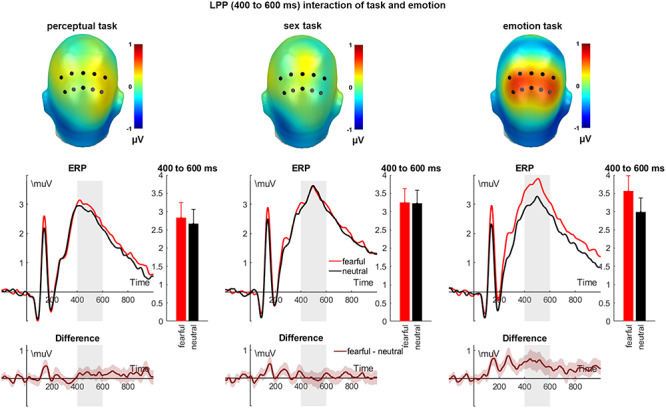
Interaction effects of emotional expression and task condition on the LPP. Scalp topographies depict the amplitude differences between fearful and neutral expressions. ERP waveforms show the time course over highlighted sensors. Respective difference plots contain 95% bootstrap confidence intervals of intra-individual differences. For bar charts, error bars show 95% confidence intervals.

For the LPP, main effects of emotion (*F*_(1,39)_ = 12.43, *P* = 0.001, η_P_^2^ = 0.242; see Figure 4) and of task were identified (*F*_(2,78)_ = 9.30, *P* < 0.001, η_P_^2^ = 0.192). Here, fearful faces elicited larger amplitudes than neutral ones. Furthermore, LPP amplitudes during the emotion and the sex task were larger than during the perceptual task (*P*s = 0.001 and 0.002), but did not differ significantly from one another (*P* = 0.711). Importantly, we observed the predicted interaction of emotion and task (*F*_(1.74,67.99)_ = 6.81, *P* = 0.003, η_P_^2^ = 0.149; see Figure 3). *Post hoc* tests show that larger emotion effects in the emotion task compared to the perceptual task (*P* = 0.015), and compared to the sex task (*P* = 0.003), with no differences between the perceptual and sex task (*P* = 0.258).

**Table 1:** Results from a 2 × 3 repeated measures ANOVA with and without reaction time (RT) as a covariate for each ERP component

	Effect	DF	DFe	ANOVA results		ANCOVA with RTs	
				*F*	*P*	*F* ^*^	*P* ^*^
P1	Emotion	1	39	0.02	0.883	0.02	0.899
	Task	2	78	0.24	0.783	0.48	0.624
	Emotion × task	2	78	2.19	0.119	2.77	0.069
N170	Emotion	1	39	**90.81**	**<0.001**	**87.66**	**<0.001**
	Task	2	78	0.91	0.405	0.79	0.460
	Emotion × task	2	78	1.73	0.184	0.77	0.465
EPN	Emotion	1	39	**14.59**	**<0.001**	**10.74**	**<0.001**
	Task	2	78	**9.37**	**<0.001**	**4.87**	**0.010**
	Emotion × task	2	78	**7.13**	**0.001**	**8.21**	**<0.001**
LPP	Emotion	1	39	**12.43**	**=0.001**	**10.51**	**0.002**
	Task	2	78	**9.30**	**<0.001**	**4.11**	**0.020**
	Emotion × task	2	78	**6.81**	**0**.**003**	**7.66**	**<0.001**

Notes: *F* and *P* columns refer to the ANOVA without covariates and F^*^ and P^*^ to the ANCOVA with reaction time as a covariate (*P*-values were Greenhouse–Geisser-corrected whenever Mauchly’s tests indicated a violation of the sphericity assumption). Significant main and interaction effects are highlighted in bold font.

### Control analyses

#### ANCOVAs with reaction time as a covariate

Since reaction time was significantly affected by the task and the interaction of emotion and task, ANCOVAs with the average reaction time for each condition as a covariate were calculated (see Table 1). For these ANCOVAs, all main effects on the N170, EPN and LPP remained significant. Further the EPN and LPP interactions of emotion and task remained significant. We found main effects slightly decreasing and interactions slightly increasing in these ANCOVAs.

#### Analyses of horizontal and vertical eye-related activity

We tested absolute activity measured by horizontal or vertical EOG channels differed for the P1, N170, EPN and LPP time windows. For horizontal EOG activity, we found no differences between emotion (*Fs*_(1,39)_ < 0.82 *P*s > 0.366) and task conditions (*Fs*_(2,78)_ < 0.97 *P*s > 0.382), and there was no interaction for all tested time window (*Fs*_(2,78)_ < 0.83 *P*s > 0.440). For vertical EOG activity, we likewise found no differences between emotion (*Fs*_(1,39)_ < 2.50 *P*s > 0.117) and task conditions (*Fs*_(2,78)_ < 1.76 *P*s > 0.175) and no interaction for all tested time windows (*Fs*_(2,78)_ < 1.80 *P*s > 0.168).

## Discussion

In this pre-registered study, we investigated how attention tasks differentially affect the emotional modulation of early, mid-latency and late ERP components towards fearful *vs* neutral faces. We found emotion effects to be task-independent for the early N170 component. At the level of the EPN, the predicted increase of emotion effects, caused by growing attention to emotionally relevant features, could be validated. Finally, in line with our predictions, LPP emotion effects were only observed when the expression itself was rendered task-relevant.

This study shows that feature-based attention does not modulate P1 and N170 effects to fearful *vs* neutral facial expressions. We predicted that emotion effects might be largest in the perceptual task, as effects are more often reported when faces serve as distracters (e.g. Santos *et al.,* 2008; Li *et al.,* 2018), or under conditions of perceptual load (e.g. Schindler *et al.,* 2019b), and therefore might indicate early inhibitory processes (Klimesch *et al.,* 2007). While we observed largest P1 amplitude differences in the perceptual task (see Figure 2), we found no significant emotion by task interaction effect. This might be due to a lack of statistical power (see Table 1) and adds to the notion that P1 emotion effects are highly variable and small in size (Schindler et al., 2019a; for a review see Schindler and Bublatzky, 2020) (reporting effects, Rellecke *et al.,* 2012; Valdés-Conroy *et al.,* 2014; reporting no effects, Itier and Neath-Tavares, 2017; Acunzo *et al.,* 2019).

For the N170, previous studies comparing different attention conditions suggest either task-insensitive (Rellecke *et al.,* 2012; Itier and Neath-Tavares, 2017) or task-modulated emotion effects (only for females: Valdés-Conroy *et al.,* 2014). In our study, we found emotion effects for fearful compared to neutral expressions, not interacting with the attention task (in line with Rellecke *et al.,* 2012; Itier and Neath-Tavares, 2017). This adds to the meta-analysis showing that fearful faces reliably potentiate N170 amplitudes (Hinojosa *et al.,* 2015). The N170 is regarded as a reflection of structural encoding and high-level face information processing, where face–object differences have frequently been reported (e.g. Rousselet *et al.,* 2008; Eimer, 2011; Rossion and Caharel, 2011; Ganis *et al.,* 2012; but see also Thierry *et al.,* 2007). Configural information appears to be of particular importance here and could be based on holistic (Piepers and Robbins, 2012; Rossion, 2013; Calvo and Beltrán, 2014) or on specific features such as the mouth (Schyns *et al.,* 2007; Harris and Nakayama, 2008; Schyns *et al.,* 2009; daSilva *et al.,* 2016) or the eye region (Schyns *et al.,* 2007; Schyns *et al.,* 2009; Itier *et al.,* 2011; Parkington and Itier, 2018). Research has found that emotional N170 modulations can be influenced by embedded context (e.g. by emotionally congruent or self-referential context; see Diéguez-Risco *et al.,* 2015; Li *et al.,* 2019), where specifically congruence between facial expressions and situational context is found to modulate this processing stage (Diéguez-Risco *et al.,* 2015; but sometimes only in interaction with task and not expression; see Aguado *et al.,* 2019). In contrast to such top-down effects, recent studies of ours showed that emotional modulations of the N170 are immune to a range of image manipulations (Schindler *et al.,* 2019a; Bruchmann *et al.,* 2020) or variations of a task’s perceptual load difficulty (Schindler *et al.,* 2019b). As the latter study did not include tasks manipulating the attention drawn to the face or the face’s emotion, our current study add to the previous findings of the N170’s insensitivity to task demands, suggesting a rather automatic extraction of emotionally relevant features.

The subsequent EPN was modulated by fearful faces, which is in line with a number of studies comparing fearful to neutral expressions (Mühlberger *et al.,* 2009; Walentowska and Wronka, 2012; Wieser *et al.,* 2012; Smith *et al.,* 2013; Schindler *et al.,* 2019a). These EPN modulations are interpreted to reflect a sensitivity to salient emotional information at this processing stage (Junghöfer *et al.,* 2001), in line with the EPN’s relation to early attentional selection processes (Schupp *et al.,* 2004; Wieser *et al.,* 2010). Emotion effects were significantly affected by an emotion × task interaction, showing increasing amplitude differences with increasing attention to emotionally relevant features. To explain these effects, we suggest that the EPN might represent a ‘bottleneck’ of elaborate emotion processing; more precisely, it might reflect a stage at which (task-oriented) attention processes compete with emotional differentiation (e.g. see Schupp *et al.,* 2007; Schindler *et al.,* 2020). This would account for the emotion effect building up with stronger attention to emotionally relevant features.

In contrast, our findings suggest that during the late processing stages, explicit attention to the expression itself is necessary to elicit differential LPP effects. This attention to the expression has recently been suggested to be crucial (Schindler *et al.,* 2019a for a review see Schindler and Bublatzky, 2020), which is supported by a study showing that LPP effects are strongest during an emotion decision task (Rellecke *et al.,* 2012). This is further in line with the postulation that larger LPP amplitudes are related to stimulus evaluation and controlled attention processes (Schupp *et al.,* 2006; Hajcak *et al.,* 2009), particularly when involving the appraisal of affective meaning (Schupp *et al.,* 2006; Wessing *et al.,* 2013). A possible underlying mechanism which is supported by our study’s results and can explain previous inconsistent LPP findings relates to the task requiring participants to deploy different aspects of feature-based attention. For late stages, we reason that top-down and bottom-up processes might interact with biologically relevant (threat-related) expressions benefitting from task relevance. Here, it might be a potentiation of an initial amygdala-dependent feedback for fearful expressions with the task relevance inducing top-down signalling from frontoparietal attention networks which synergistically increases threat-related processing (Pourtois *et al.,* 2013). Such processes are vulnerable to competing tasks (e.g. see Schupp *et al.,* 2007). Of note, we observed reaction time differences between task conditions, but also differently across the tasks between fearful and neutral faces. While we used ANCOVAs correcting ERP effects with respective conditional reaction times, there might be a trial-wise influence on ERP modulations and RTs which cannot be addressed by these analyses (see the limitations section).

Our study provides findings which can be integrated into a comprehensive model of facial emotion perception as a function of attention. Building on recent models of face processing (Haxby and Gobbini, 2011; Schweinberger and Neumann, 2016), specialized systems are suggested for the processing of basic visual facial features and for extended functions such as emotion processing and allocation of attentional resources. Importantly, the interplay of these systems flexibly varies across the visual processing stream, which finds its reflection in subsequent and partly overlapping ERP correlates. At the P1 time window, low-level analysis takes place, followed by configural face analyses during the N170 window. For the EPN, early attention processes integrate low-level information and task-relevant features, and at the stage of the LPP, expression differentiation is enhanced by relevance—this also includes evaluative, episodic, personal and biographical information available for the presented faces (see Schweinberger and Neumann, 2016; Schindler and Bublatzky, 2020).

Some further remarks shall be made on the behavioural responses: While we observed a ceiling effect for accuracy values, we found interactions regarding reaction times. No differences between fearful and neutral faces were found when attending to line orientation, but faster responses to fearful faces were made in the sex discrimination task, and slower responses in the emotion task. This fits recent findings highlighting the impact of task focus on reaction time differences and showing that, for example, a differentiation between fear- and anger-related words occurs only when approach-withdrawal decisions are focused (Huete-Pérez *et al.,* 2019).

### Constraints on generality

With regard to our study’s findings, there are some constraints which have to be mentioned. Our sample contained mostly female participants, and generalizing our findings to males should be taken with care since previous work has suggested sex differences with respect to the processing of emotional information (for a review, see Kret and De Gelder, 2012). Regarding ERPs, women exhibit larger mid-latency modulations for emotional vocalizations (Schirmer *et al.,* 2019) and larger LPP responses to images conveying interpersonal touch in implicit tasks (Schirmer and McGlone, 2019) and show task-modulated N170 emotion effects (Valdés-Conroy *et al.,* 2014). Furthermore, each emotional expression was repeated 6 times and each identity even 12 times in total. We used a homogenous stimulus to control for visual differences which have been shown to influence differential emotional modulations (displayed teeth, see daSilva *et al.,* 2016; emotion-specific frequencies, see Bruchmann *et al.,* 2020). While studies using pictorial scenes found this number of repetitions not altering differential emotion effects (Olofsson and Polich, 2007; Ferrari *et al.,* 2013; for a recent review, see Ferrari *et al.,* 2017), it is unclear if this also applies to (early) ERPs for emotional expressions. Furthermore, we found that interactions effects remained significant when using ANCOVAs with reaction times. However, we cannot exclude influences of single trials on our ERPs, and our findings need to be replicated with matched difficulty. This requires intense piloting to result in similar reaction times for classifying fearful and neutral expressions across the tasks. Finally, studies examining visual attention rely on eye-movement rejection to avoid condition differences on blinks or saccades (e.g. recommended by Luck, 2014). We pre-registered to use eye-movement correction to obtain a minimum number of trials per cell and examined average horizontal and vertical EOG activity. While we find no statistical differences, we cannot exclude influences of eye-related activity on ERPs. Further studies are needed with higher numbers of trials to use an eye-movement rejection approach and replicate our findings with matched task difficulty, also clarifying the influence of identity repetitions and participants’ sex on the current ERP findings.

## Conclusion

To summarize, this study shows that early N170 emotion effects are task-independent while EPN and LPP effects depend on the attended feature. These findings are vitally important for researchers who conduct ERP studies using facial expressions as they reveal a systematic pattern of emotional sensitivity varying with competing attention tasks and therefore enable the formulation of clear predictions.

## References

[ref1] Acunzo D., MacKenzie G., Rossum M.C.W. van (2019). Spatial attention affects the early processing of neutral versus fearful faces when they are task-irrelevant: a classifier study of the EEG C1 component. Cognitive, Affective, & Behavioral Neuroscience, 19, 123–37. 10.3758/s13415-018-00650-7 30341623

[ref2] Aguado L., Parkington K.B., Dieguez-Risco T., et al. (2019). Joint modulation of facial expression processing by contextual congruency and task demands. Brain Sciences, 9, 116. 10.3390/brainsci9050116 PMC6562852 31109022

[ref3] Bruchmann M., Schindler S., Straube T. (2020). The spatial frequency spectrum of fearful faces modulates early and mid-latency ERPs but not the N170. Psychophysiology, n/a, e13597. 10.1111/psyp.13597 32390215

[ref4] Busch N.A., Debener S., Kranczioch C., et al. (2004). Size matters: effects of stimulus size, duration and eccentricity on the visual gamma-band response. Clinical Neurophysiology, 115, 1810–20. 15261860 10.1016/j.clinph.2004.03.015

[ref5] Calvo M.G., Beltrán D. (2014). Brain lateralization of holistic versus analytic processing of emotional facial expressions. NeuroImage, 92, 237–47. 24495810 10.1016/j.neuroimage.2014.01.048

[ref6] daSilva E.B., Crager K., Geisler D., et al. (2016). Something to sink your teeth into: the presence of teeth augments ERPs to mouth expressions. NeuroImage, 127, 227–41. 26706446 10.1016/j.neuroimage.2015.12.020

[ref7] Diéguez-Risco T., Aguado L., Albert J., et al. (2015). Judging emotional congruency: explicit attention to situational context modulates processing of facial expressions of emotion. Biological Psychology, 112, 27–38. 26450006 10.1016/j.biopsycho.2015.09.012

[ref8] Eimer M. (2011). The face-sensitive N170 component of the event-related brain potential In: Gillian Rhodes, Andy Calder, Mark Johnson, and James V. Haxby, editors. The Oxford Handbook of Face Perception, Oxford: Oxford University Press (OUP), 28, pp. 329–44. doi: 10.1093/oxfordhb/9780199559053.013.0017.

[ref9] Faul F., Erdfelder E., Buchner A., et al. (2009). Statistical power analyses using G^*^ power 3.1: tests for correlation and regression analyses. Behavior Research Methods, 41, 1149–60. 19897823 10.3758/BRM.41.4.1149

[ref10] Ferrari V., Bradley M.M., Codispoti M., et al. (2013). Repetition and brain potentials when recognizing natural scenes: task and emotion differences. Social Cognitive and Affective Neuroscience, 8, 847–54. 22842817 10.1093/scan/nss081 PMC3831551

[ref11] Ferrari V., Codispoti M., Bradley M.M. (2017). Repetition and ERPs during emotional scene processing: a selective review. International Journal of Psychophysiology, 111, 170–7. 27418540 10.1016/j.ijpsycho.2016.07.496

[ref12] Foti D., Olvet D.M., Klein D.N., et al. (2010). Reduced electrocortical response to threatening faces in major depressive disorder. Depression and Anxiety, 27, 813–20. 20577985 10.1002/da.20712

[ref13] Framorando D., Burra N., Bapst M., et al. (2018). ERP responses greater for faces in the temporal compared to the nasal visual field. Neuroscience Letters, 665, 7–12. 29155351 10.1016/j.neulet.2017.11.031

[ref14] Ganis G., Smith D., Schendan H.E. (2012). The N170, not the P1, indexes the earliest time for categorical perception of faces, regardless of interstimulus variance. NeuroImage, 62, 1563–74. 22634853 10.1016/j.neuroimage.2012.05.043

[ref15] Grunewald M., Döhnert M., Brandeis D., et al. (2019). Attenuated LPP to emotional face stimuli associated with parent- and self-reported depression in children and adolescents. Journal of Abnormal Child Psychology, 47, 109–18. 29679244 10.1007/s10802-018-0429-3

[ref16] Hajcak G., Dunning J.P., Foti D. (2009). Motivated and controlled attention to emotion: time-course of the late positive potential. Clinical Neurophysiology: Official Journal of the International Federation of Clinical Neurophysiology, 120, 505–10. 19157974 10.1016/j.clinph.2008.11.028

[ref17] Harris A., Nakayama K. (2008). Rapid adaptation of the M170 response: importance of face parts. Cerebral Cortex, 18, 467–76. 17573371 10.1093/cercor/bhm078

[ref18] Hautzinger M., Keller F., Kühner C. (2009). BDI-II. Beck-Depressions-Inventar. Revision, Frankfurt/Main: Pearson Assessment.

[ref19] Haxby J.V., Gobbini M.I. (2011). Distributed Neural Systems for Face Perception, The Oxford Handbook of Face Perception. Oxford Handbook of Face Perception, Edited by Gillian Rhodes, Andy Calder, Mark Johnson, and James V. Haxby, Oxford University Press (OUP), Oxford, doi: 10.1093/oxfordhb/978019955.9053.013.0017.

[ref20] Herbert C., Sfaerlea A., Blumenthal T. (2013). Your emotion or mine: labeling feelings alters emotional face perception—an ERP study on automatic and intentional affect labeling. Frontiers in Human Neuroscience, 7, 1–14. 23888134 10.3389/fnhum.2013.00378 PMC3719026

[ref21] Hinojosa J.A., Mercado F., Carretié L. (2015). N170 sensitivity to facial expression: a meta-analysis. Neuroscience and Biobehavioral Reviews, 55, 498–509. 26067902 10.1016/j.neubiorev.2015.06.002

[ref22] Hopfinger J.B., Mangun G.R. (1998). Reflexive attention modulates processing of visual stimuli in human extrastriate cortex. Psychological Science, 9, 441–7. 26321798 10.1111/1467-9280.00083 PMC4552358

[ref23] Huete-Pérez D., Haro J., Hinojosa J.A., et al. (2019). Does it matter if we approach or withdraw when reading? A comparison of fear-related words and anger-related words. Acta Psychologica, 197, 73–85. 31125899 10.1016/j.actpsy.2019.04.018

[ref24] Ille N., Berg P., Scherg M. (2002). Artifact correction of the ongoing EEG using spatial filters based on artifact and brain signal topographies. Journal of Clinical Neurophysiology: Official Publication of the American Electroencephalographic Society, 19, 113–24. 11997722 10.1097/00004691-200203000-00002

[ref25] Itier R.J., Neath-Tavares K.N. (2017). Effects of task demands on the early neural processing of fearful and happy facial expressions. Brain Research, 1663, 38–50. 28315309 10.1016/j.brainres.2017.03.013 PMC5756067

[ref26] Itier R.J., Van Roon P., Alain C. (2011). Species sensitivity of early face and eye processing. NeuroImage, 54, 705–13. 20650321 10.1016/j.neuroimage.2010.07.031 PMC3933319

[ref27] Junghöfer M., Bradley M.M., Elbert T.R., et al. (2001). Fleeting images: a new look at early emotion discrimination. Psychophysiology, 38, 175–8. 11347862

[ref28] Keil A., Debener S., Gratton G., et al. (2014). Committee report: publication guidelines and recommendations for studies using electroencephalography and magnetoencephalography. Psychophysiology, 51, 1–21. 24147581 10.1111/psyp.12147

[ref29] Klimesch W., Sauseng P., Hanslmayr S. (2007). EEG alpha oscillations: the inhibition–timing hypothesis. Brain Research Reviews, 53, 63–88. 16887192 10.1016/j.brainresrev.2006.06.003

[ref30] Körner A., Geyer M., Roth M., et al. (2008). Persönlichkeitsdiagnostik mit dem neo-fünf-faktoren-inventar: die 30-item-kurzversion (neo-ffi-30). Psychotherapie Psychosomatik· Medizinische Psychologie, 58, 238–45. 10.1055/s-2007-986199 17899495

[ref31] Kret M.E., De Gelder B. (2012). A review on sex differences in processing emotional signals. Neuropsychologia, 50, 1211–21. 22245006 10.1016/j.neuropsychologia.2011.12.022

[ref32] Langner O., Dotsch R., Bijlstra G., et al. (2010). Presentation and validation of the Radboud faces database. Cognition and Emotion, 24, 1377–88.

[ref33] Lawrence M.A., Lawrence M.M.A. (2016). Package ‘ez’. R package version, 4(0).

[ref34] Li Q., Zhou S., Zheng Y., et al. (2018). Female advantage in automatic change detection of facial expressions during a happy-neutral context: an ERP study. Frontiers in Human Neuroscience, 12, 1–10.. 29725293 10.3389/fnhum.2018.00146 PMC5917044

[ref35] Li S., Zhu X., Ding R., et al. (2019). The effect of emotional and self-referential contexts on ERP responses towards surprised faces. Biological Psychology, 146, 107728. 31306692 10.1016/j.biopsycho.2019.107728

[ref36] Liu Y., Huang H., McGinnis-Deweese M., et al. (2012). Neural substrate of the late positive potential in emotional processing. The Journal of Neuroscience, 32, 14563–72. 23077042 10.1523/JNEUROSCI.3109-12.2012 PMC3516184

[ref37] Luck S.J. (2014). An Introduction to the Event-related Potential Technique, Cambridge, MA: MIT Press.

[ref38] Luck S.J., Hillyard S.A. (1994). Electrophysiological correlates of feature analysis during visual search. Psychophysiology, 31, 291–308. 8008793 10.1111/j.1469-8986.1994.tb02218.x

[ref39] MacNamara A., Ferri J., Hajcak G. (2011). Working memory load reduces the late positive potential and this effect is attenuated with increasing anxiety. Cognitive, Affective, & Behavioral Neuroscience, 11, 321–31. 10.3758/s13415-011-0036-z 21556695

[ref40] Mühlberger A., Wieser M.J., Herrmann M.J., et al. (2009). Early cortical processing of natural and artificial emotional faces differs between lower and higher socially anxious persons. Journal of Neural Transmission, 116, 735–46. 18784899 10.1007/s00702-008-0108-6

[ref41] Müller-Bardorff M., Schulz C., Peterburs J., et al. (2016). Effects of emotional intensity under perceptual load: an event-related potentials (ERPs) study. Biological Psychology, 117, 141–9. 26995785 10.1016/j.biopsycho.2016.03.006

[ref42] Müller-Bardorff M., Bruchmann M., Mothes-Lasch M., et al. (2018). Early brain responses to affective faces: a simultaneous EEG-fMRI study. NeuroImage, 178, 660–7. 29864521 10.1016/j.neuroimage.2018.05.081

[ref43] Neath-Tavares K.N., Itier R.J. (2016). Neural processing of fearful and happy facial expressions during emotion-relevant and emotion-irrelevant tasks: a fixation-to-feature approach. Biological Psychology, 119, 122–40. 27430934 10.1016/j.biopsycho.2016.07.013 PMC5319862

[ref44] Nolan H., Whelan R., Reilly R.B. (2010). FASTER: fully automated statistical thresholding for EEG artifact rejection. Journal of Neuroscience Methods, 192, 152–62. 20654646 10.1016/j.jneumeth.2010.07.015

[ref45] Olofsson J.K., Polich J. (2007). Affective visual event-related potentials: arousal, repetition, and time-on-task. Biological Psychology, 75, 101–8. 17275979 10.1016/j.biopsycho.2006.12.006 PMC1885422

[ref46] Parkington K.B., Itier R.J. (2018). One versus two eyes makes a difference! Early face perception is modulated by featural fixation and feature context. Cortex, 109, 35–49. 30286305 10.1016/j.cortex.2018.08.025

[ref47] Peltola M.J., IJzendoorn M.H. van, Yrttiaho S. (2018). Attachment security and cortical responses to fearful faces in infants. Attachment & Human Development, 0, 1–15. 10.1080/14616734.2018.1530684 30304989

[ref48] Peyk P., De Cesarei A., Junghöfer M. (2011). Electro magneto encephalograhy software: overview and integration with other EEG/MEG toolboxes. Computational Intelligence and Neuroscience, 2011, 1–11 Article ID 861705. 21577273 10.1155/2011/861705 PMC3090751

[ref49] Piepers D., Robbins R. (2012). A review and clarification of the terms “holistic,” “configural,” and “relational” in the face perception literature. Frontiers in Psychology, 3, 1–11. 23413184 10.3389/fpsyg.2012.00559 PMC3571734

[ref50] Pourtois G., Schettino A., Vuilleumier P. (2013). Brain mechanisms for emotional influences on perception and attention: what is magic and what is not. Biological Psychology, 92, 492–512. 22373657 10.1016/j.biopsycho.2012.02.007

[ref51] Rellecke J., Sommer W., Schacht A. (2012). Does processing of emotional facial expressions depend on intention? Time-resolved evidence from event-related brain potentials. Biological Psychology, 90, 23–32. 22361274 10.1016/j.biopsycho.2012.02.002

[ref52] Rossion B. (2013). The composite face illusion: a whole window into our understanding of holistic face perception. Visual Cognition, 21, 139–253.

[ref53] Rossion B., Caharel S. (2011). ERP evidence for the speed of face categorization in the human brain: disentangling the contribution of low-level visual cues from face perception. Vision Research, 51, 1297–311. 21549144 10.1016/j.visres.2011.04.003

[ref54] Rousselet G.A., Husk J.S., Bennett P.J., et al. (2008). Time course and robustness of ERP object and face differences. Journal of Vision, 8, 3–3. 10.1167/8.12.3 18831616

[ref55] Sabatinelli D., Lang P.J., Keil A., et al. (2007). Emotional perception: correlation of functional MRI and event-related potentials. Cerebral Cortex, 17, 1085–91 Epub 12 June 2006. 16769742 10.1093/cercor/bhl017

[ref56] Sabatinelli D., Frank D.W., Wanger T.J., et al. (2014). The timing and directional connectivity of human frontoparietal and ventral visual attention networks in emotional scene perception. Neuroscience, 277, 229–38. 25018086 10.1016/j.neuroscience.2014.07.005

[ref57] Santos I.M., Iglesias J., Olivares E.I., et al. (2008). Differential effects of object-based attention on evoked potentials to fearful and disgusted faces. Neuropsychologia, 46, 1468–79. 18295286 10.1016/j.neuropsychologia.2007.12.024

[ref58] Schindler S., Zell E., Botsch M., et al. (2017). Differential effects of face-realism and emotion on event-related brain potentials and their implications for the uncanny valley theory. Scientific Reports, 7, 45003. 28332557 10.1038/srep45003 PMC5362933

[ref59] Schindler S., Bruchmann M., Bublatzky F., et al. (2019a). Modulation of face- and emotion-selective ERPs by the three most common types of face image manipulations. Social Cognitive and Affective Neuroscience, 14, 493–503. 30972417 10.1093/scan/nsz027 PMC6545565

[ref60] Schindler S., Bruchmann M., Gathmann B., et al. (2019b). Effects of low-level visual information and perceptual load on P1 and N170 responses to emotional faces. PsyArxiv. doi: 10.31234/osf.io/3rd5z. 33450599

[ref61] Schindler S., Caldarone F., Bruchmann M., et al. (2020). Time-dependent effects of perceptual load on processing fearful and neutral faces. Neuropsychologia, 146, 107529, 1–10. 10.1016/j.neuropsychologia.2020.107529 32553724

[ref61a] Schindler, S., Bublatzky, F. (2020). Attention and emotion: An integrative review of emotional face processing as a function of attention. Cortex. 10.1016/j.cortex.2020.06.010. 32745728

[ref62] Schirmer A., McGlone F. (2019). A touching sight: EEG/ERP correlates for the vicarious processing of affectionate touch. Cortex, 111, 1–15. 30419352 10.1016/j.cortex.2018.10.005

[ref63] Schirmer A., Wijaya M., Wu E., et al. (2019). Vocal threat enhances visual perception as a function of attention and sex. Social Cognitive and Affective Neuroscience, 14, 727–35. 31216037 10.1093/scan/nsz044 PMC6778830

[ref64] Schupp H.T., Junghöfer M., Weike A.I., et al. (2004). The selective processing of briefly presented affective pictures: an ERP analysis. Psychophysiology, 41, 441–9. 15102130 10.1111/j.1469-8986.2004.00174.x

[ref65] Schupp H.T., Flaisch T., Stockburger J., et al. (2006). Chapter 2. Emotion and attention: event-related brain potential studies. Progress in Brain Research, 156, 31–51. 17015073 10.1016/S0079-6123(06)56002-9

[ref66] Schupp H.T., Stockburger J., Bublatzky F., et al. (2007). Explicit attention interferes with selective emotion processing in human extrastriate cortex. BMC Neuroscience, 8, 16. 17316444 10.1186/1471-2202-8-16 PMC1808466

[ref67] Schweinberger S.R., Neumann M.F. (2016). Repetition effects in human ERPs to faces. Cortex, 80, 141–53. 26672902 10.1016/j.cortex.2015.11.001

[ref68] Schyns P.G., Petro L.S., Smith M.L. (2007). Dynamics of visual information integration in the brain for categorizing facial expressions. Current Biology, 17, 1580–5. 17869111 10.1016/j.cub.2007.08.048

[ref69] Schyns P.G., Petro L.S., Smith M.L. (2009). Transmission of facial expressions of emotion co-evolved with their efficient decoding in the brain: behavioral and brain evidence. PLoS One, 4, e5625. 19462006 10.1371/journal.pone.0005625 PMC2680487

[ref70] Smith E., Weinberg A., Moran T., et al. (2013). Electrocortical responses to NIMSTIM facial expressions of emotion. International Journal of Psychophysiology, 88, 17–25. 23280304 10.1016/j.ijpsycho.2012.12.004

[ref71] Spielberger C.D., Sydeman S.J., Owen A.E., et al. (1999). Measuring anxiety and anger with the State-Trait Anxiety Inventory (STAI) and the State-Trait Anger Expression Inventory (STAXI) In: Maruish M.E., editor. The Use of Psychological Testing for Treatment Planning and Outcomes Assessment, 2nd edn, Lawrence Erlbaum Associates: Mahwah, pp. 993–1021.

[ref72] Syrjänen E., Wiens S., Fischer H., et al. (2018). Background odors modulate N170 ERP component and perception of emotional facial stimuli. Frontiers in Psychology, 9, 1–11. 29997539 10.3389/fpsyg.2018.01000 PMC6029154

[ref73] Thierry G., Martin C.D., Downing P., et al. (2007). Controlling for interstimulus perceptual variance abolishes N170 face selectivity. Nature Neuroscience, 10, 505–11. 17334361 10.1038/nn1864

[ref74] Valdés-Conroy B., Aguado L., Fernández-Cahill M., et al. (2014). Following the time course of face gender and expression processing: a task-dependent ERP study. International Journal of Psychophysiology, 92, 59–66. 24594443 10.1016/j.ijpsycho.2014.02.005

[ref75] Vogel E.K., Luck S.J. (2000). The visual N1 component as an index of a discrimination process. Psychophysiology, 37, 190–203. 10731769

[ref76] Walentowska W., Wronka E. (2012). Trait anxiety and involuntary processing of facial emotions. International Journal of Psychophysiology, 85, 27–36. 22210124 10.1016/j.ijpsycho.2011.12.004

[ref77] Wessing I., Rehbein M.A., Postert C., et al. (2013). The neural basis of cognitive change: reappraisal of emotional faces modulates neural source activity in a frontoparietal attention network. NeuroImage, 81, 15–25. 23664945 10.1016/j.neuroimage.2013.04.117

[ref78] Wieser M.J., Pauli P., Reicherts P., et al. (2010). Don’t look at me in anger! Enhanced processing of angry faces in anticipation of public speaking. Psychophysiology, 47, 271–80. 20030758 10.1111/j.1469-8986.2009.00938.x

[ref79] Wieser M.J., Gerdes A.B.M., Greiner R., et al. (2012). Tonic pain grabs attention, but leaves the processing of facial expressions intact—evidence from event-related brain potentials. Biological Psychology, 90, 242–8. 22503790 10.1016/j.biopsycho.2012.03.019

[ref80] Wu L., Müller H.J., Zhou X., et al. (2019). Differential modulations of reward expectation on implicit facial emotion processing: ERP evidence. Psychophysiology, 56, e13304. 30443953 10.1111/psyp.13304

